# Aggravated Risks of Emergency Hospitalizations Associated with Temperature amid Elevated Ambient Air Pollution: Evidence from a 20-Year Time-Series Study in Hong Kong

**DOI:** 10.1021/acs.est.5c10594

**Published:** 2025-12-10

**Authors:** Yi Tong Guo, Yingxin Li, Kin Fai Ho, Ka Hung Chan

**Affiliations:** † JC School of Public Health and Primary Care, 26451 The Chinese University of Hong Kong, Hong Kong SAR, China; ‡ Institute of Environment, Energy and Sustainability, The Chinese University of Hong Kong, Hong Kong SAR, China; § Oxford Global Health, 6396 University of Oxford, Oxford OX37LF, U.K.; ∥ Clinical Trial Service Unit and Epidemiological Studies Unit, Nuffield Department of Population Health, University of Oxford, Oxford OX37LF, U.K.

**Keywords:** temperature, air pollution, PM_2.5_, NO_2_, O_3_, modification, hospitalization

## Abstract

The combined impact of nonoptimal temperatures and air pollution on hospitalizations remains understudied. This study investigated how major criterion air pollutants (PM_2.5_, NO_2_, and O_3_) modified the short-term temperature–hospitalization associations and quantified the excess attributable burden. Daily time-series data of emergency hospitalizations and environmental exposures (2000–19) in Hong Kong were analyzed using two comparative models with product terms (1) between temperature and lower and higher strata for pollutants, and (2) between temperature and the count of elevated pollutants. Over 10 million noncancer nonexternal (NCNE) admissions occurred, including ∼14% circulatory and ∼20% respiratory admissions. PM_2.5_ consistently amplified temperature effects, leading to 1.5–2.6% significantly higher NCNE risks with low and high temperatures and 2.8–3.7% higher circulatory risks with low temperatures. NO_2_ also intensified circulatory risks. Temperature effects were 1.4–2.6% higher on polluted days (≥1 elevated pollutants) than on unpolluted days (0 elevated pollutants), with positive linear trends as pollutant counts increased (p-trend <0.05). The observed temperature-attributable numbers (fraction, %) without considering pollution modification were 68,032 (0.7%), 44,290 (3.2%), and 39,995 (2.0%) for NCNE, circulatory, and respiratory admissions, respectively. These could be reduced by 0.5–0.8% in counterfactual low-pollution scenarios. Combined air pollution exposure exacerbates temperature-related hospitalization risks in Hong Kong, particularly for NCNE and circulatory admissions.

## Graphical abstract

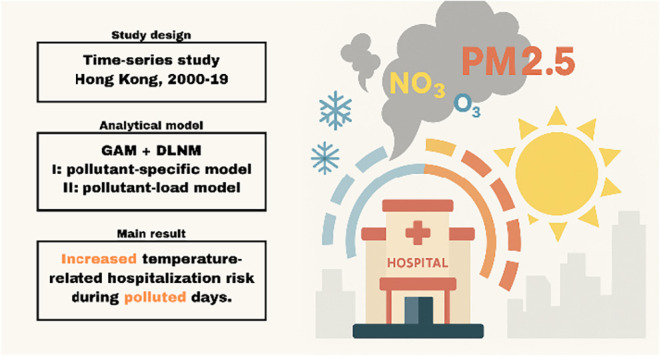

## Introduction

Unprecedented urbanization and industrialization have emerged as a pivotal force driving climate change and contributing to environmental pollution challenges globally.^1^ Major air pollutants such as particulate matter (PM), ozone (O_3_), and nitrogen oxides (NO_*x*_) remain at harmful levels worldwide, with >99% the global population exposed to concentrations exceeding the World Health Organization (WHO) guidelines.^2^ Both nonoptimal temperatures (i.e., cold or heat) and ambient air pollution have been well-documented risk factors for premature mortality and a variety of diseases.^3–6^

People rarely experience environmental hazards in isolation. Traditionally, studies on the health effects of temperature and air pollution have treated one as a confounder for the other, overlooking their potential combined effects.^7,8^ However, emerging evidence indicates effect modification or synergism between them, with more consistent findings for heat and PM in relation to mortality risk.^9–13^ For example, a meta-analysis by Hu et al. (2022) found that PM_10_ and O_3_ significantly amplified heat-related mortality risks but not those associated with heatwaves or cold.^9^ Three reviews suggested high temperatures and warm seasons enhanced pollutant-related mortality and morbidity, especially for cardiorespiratory outcomes, whereas cold modification was inconsistent.^10–12^ Biologically, temperature and pollutants may interact through shared pathways involving systemic inflammation, oxidative stress, endothelial dysfunction, and autonomic imbalance.^13^

However, research on the impact of combined exposure to temperature and air pollution on hospitalizations is limited, with most investigating temperature or seasonal modification of pollutant-health associations. Pollutant effects, particularly those of PM, were found to amplify during cold conditions in some U.S. and Asian cities,^14–18^ while some other studies reported larger risks during warm conditions^19–21^ or no clear modification.^22,23^ Conversely, far fewer studies have examined pollutants as modifiers for temperature effects. In Brisbane, Australia, elevated PM_10_ offsets the otherwise protective effects of warmer temperatures on cardiorespiratory hospitalizations.^24^ In New England, high PM_2.5_ intensified adverse temperature effects on respiratory admissions but weakened those on cardiac admissions in older adults.^25^ In China, studies conducted in Shenzhen and Hefei reported stronger cold-related risks for stroke and childhood asthma hospitalizations under high PM conditions, with PM_2.5_ exerting a greater modifying effect than PM_10_.^26,27^ These studies suggest pollutant modification of temperature effects is evident but context-dependent, underscoring the need for further research across climate conditions, urban development status, and disease outcomes.

Hence, this study aimed to investigate how major criteria air pollutants—PM_2.5_, NO_2_, and O_3_—modify the short-term associations between nonoptimal temperatures and emergency hospitalizations in Hong Kong, a densely populated subtropical metropolis with unique climatic and pollution patterns. Notably, we examined modification by a specific pollutant and by pollution loads due to multiple pollutants reaching alarming levels simultaneously.

## Materials and Methods

### Study Population

Hong Kong is a densely populated urban city of 7.8 million people in southern China. With a subtropical climate, summer months of June–September are typically hot and humid when afternoon temperatures often surpass 31 °C; winters are mild and drier with temperatures between 15 and 20 °C.^28^ Hong Kong’s air quality endures challenges from local street-level pollution and regional smog.^29^ Over the past decade, criteria air pollutants like PM, NO_2_, and sulfur dioxide (SO_2_) have decreased by 37–58%, while O_3_ has been rising.^29^ However, their levels have yet to meet the Air Quality Guidelines of the WHO.^29^

### Data Collection

#### Emergency Hospitalization Data

We obtained daily counts of hospitalizations via the accident and emergency department for 2000–2019 from the Hospital Authority, which provides ∼90% of total health services in Hong Kong.^30^ The cause groups of interest, defined according to the International Classification of Diseases, Ninth Revision, Clinical Modification code (ICD-9-CM), include noncancer nonexternal (NCNE; ICD-9-CM: 001–139, 240–799), circulatory (390–459), and respiratory (460–519). Daily counts of 1–4 hospitalizations (<0.1% of total records) were masked by the Hospital Authority for reidentification concerns and imputed as 4; given their low frequency, there is no reason to expect this approach to affect the results.

#### Meteorological Data

Daily levels of mean ambient temperature (°C), relative humidity (RH, %), total rainfall (mm), and mean wind speed (km/h), were obtained for the same period from the Hong Kong Observatory Headquarter in the city center (Tsim Sha Tsui area), which has been commonly used in prior research to represent the citywide exposure.^31,32^ Missing values in wind speed (∼1.4%) were imputed by the 5-day moving averages, following a commonly adopted approach in environmental time-series studies to minimize bias while retaining temporal variation.^33^

#### Air Pollutant Data

Daily mean levels of three criteria air pollutants (in μg/m^3^), including PM_2.5_, NO_2_, and O_3_, were obtained from the Environmental Protection Department, which manages an air quality monitoring network of 14 general stations and 3 roadside stations across the city. We averaged pollutant data from 13 general stations to date, excluding the Tap Mun station located in a mountainous area.

### Statistical Analysis

Summary statistics and pairwise Pearson correlation coefficients were calculated for hospitalizations and environmental exposures. Levels of rainfall, wind speed, and air pollutants were log-transformed to correct for skewness.

Initially, we fitted quasi-Poisson generalized additive models (GAM) plus distributed lag nonlinear models (DLNM) to examine the individual effects of temperature and air pollutants, respectively, adjusting for environmental covariates and temporal variation (thereafter refer to as “raw models” without interaction term with air pollution, see eq 1).^34,35^ DLNMs allow simultaneous modeling of nonlinear exposure–response shapes and lagged effects, and are therefore appropriate for temperature, for which the health impacts have been shown to be both nonlinear and delayed.^3,4^ In contrast, pollutants were modeled linearly, given evidence of largely linear and immediate associations.^36^

$$Y_{t}∼quasi\;poisson\left(\mu_{t}\right),\log\left(\mu_{t}\right)=c{\mathrm{bt}}_{t,l}+{p_{t}+RH}_{t}+{wind}_{t}+{rain}_{t}+s\left(DOS_{t},k\right)+DOW_{t}+holiday_{t}+\mu_{t-1}+\mu_{t-2}$$ 1

Here, μ_*t*_ was the expected count of hospitalizations (*Y*) on day *t*. The crossbasis function cbt_t,l_ was created using DLNM for temperature with a maximum lag (*l*) of 7 days.^35^ We used natural cubic splines with two knots at the 10th and 90th percentiles of the distribution for the exposure dimension and quadratic B-splines with two knots equally spaced on the log scale of the lag range for the lag dimension.^37^ Pollutant levels (*p*_t_) were modeled linearly and individually, with multiple moving average (MA) windows tested, including 0 (same day), 01, 02, and 03 days. As MA03 showed the largest effects for most pollutants, it was selected for the main analysis. All models were adjusted for the following covariates (denoted as COV_t_ in the subsequent equations): same-day RH (RH_t_), wind speed (Wind_t_), and total rainfall (Rain_t_) as they might affect perceived temperature or prevent hospital visits among patients with mild symptoms;^38^ thin-plate splines of days of the study [*s*(DOS_t_)] with the number of basis (*k*) ranging from 4 to 8/year to control for trend and seasonality, day of the week (DOW_t_), public holidays (Holiday_t_), and at most two autoregression terms (μ_t‑1_ and μ_t‑2_) to reduce correlation between adjacent days. The choices of *k* and number of autoregression terms were determined by the least quasi-Akaike’s Information Criterion (qAIC) scores and residual partial autocorrelation.^34^

To determine whether air pollutants modify the temperature–hospitalization associations, we developed two separate models involving product terms: (1) between temperature and pollutant strata, which was a binary indicator of lower and higher levels of a specific pollutant (hereafter referred to as “pollutant-specific models,” see eq 2), and (2) between temperature and overall pollution load, represented by the number of pollutants at higher strata on the same day (hereafter referred to as “pollutant-load models,” see eq 3).

2.1 $$\log\left(\mu_{t}\right)=c{bt}_{t,l}+p_{t}+{cbt}_{t,l}\times P_{t}+{\mathrm{Cov}}_{t}$$

$$\log\left(\mu_{t}\right)=c{bt}_{t,l}+{cbt}_{t,l}\times P_{load,t}+{\mathrm{Cov}}_{t}$$ 2.2

In eq 2, the pollutant strata indicator *P*_t_ was derived from *p*_t_, classified as “higher” if *p*_t_ exceeded a specified percentile threshold and as “lower” otherwise. We evaluated thresholds at the 25th, 33rd, 50th, 66th, 75th, and 85th percentiles (Table S1) to identify the potential critical threshold of detecting modification, given the absence of established cutoffs in prior studies. Assessing multiple thresholds also enabled a sensitivity analysis to test the robustness of our results. Several of these percentiles also approximate WHO Air Quality Guideline concentrations (e.g., PM_2.5_ 24-h target 25 μg/m^3^), allowing us to highlight health risks at levels near or above recommended standards. *P*_*t*_ was then multiplied with cbt_t,l_ to form an interaction product. In eq 3, the pollutant-load indicator *P*_load,t_ in the product term reflected the number of pollutants at their higher strata on the same day, initially classified as “unpolluted days” when the count was 0 and ″polluted days” when the count was 1–3. Additionally, we examined *P*_load,t_ with counts of 0, 1, 2, and 3 for potential linear trends in temperature effects with increasing numbers of pollutants.

Low and high temperatures were defined as the 5th (14.6 °C) and 95th (30.0 °C) percentiles of daily mean temperature, a conventional choice to capture relatively local extremes but sufficiently frequent exposures.^39,40^ Relative risks (RR) and 95% confidence intervals (CI) for hospitalizations at these temperatures, relative to the median (24.8 °C), were calculated across different *P*_*t*_ and *P*_load,t_, by summing up the main effect from cbt_t,l_ and the extra effect from the interaction products, following standard rules for the expected value and variance of the sum of two random variables.^41^ The ratios of relative risks (RRR) and 95% CIs were calculated as RR_higher_/RR_lower_ for pollutant-specific models and RR_polluted_/RR_unpolluted_ for pollutant-load models using the Altman method.^42^ For pollutant-load models with a 4-level *P*_load,t_, we calculated the p-value for trend using meta-regression with inverse-variance weighting.^43^

Furthermore, we estimated the total number (AN) and fraction (AF, %) of hospitalizations attributable to nonoptimal temperatures based on RRs from the raw, pollutant-specific, and pollutant-load models using the following equations, as in a previous study^44^

3.1 $$AN=\sum_{t=1}^{7305}n_{t}\cdot \left({\mathrm{RR}}_{t}-1\right)$$

3.2 $$AF=\frac{AN}{\sum_{t=1}^{7305}n_{t}}\times 100\%$$

with *n*_t_ as the observed count of hospitalizations and *RR*_t_ as the overall cumulative RR corresponding to the temperature on day *t*. We computed 95% empirical confidence intervals (eCI) using 3000 Monte Carlo simulations.^44^ While the raw models reflected the observed burden of nonoptimal temperatures without accounting for pollution modification, pollutant-specific and -load models captured three scenarios: (1) “real-life scenario,” applying strata-/load-specific RRs for each day’s observed pollution; (2) “low-pollution scenario,” applying lower-strata/unpolluted RRs for all days; and (3) “high-pollution scenario,” applying higher-strata/polluted RRs for all days, with the latter two representing counterfactual scenarios at universally low and high-pollution settings.

In addition to testing multiple percentile thresholds of air pollutants, we performed several sensitivity analyses to assess the robustness of the results, including: (1) adjusting for three pollutants at the same time, (2) using MA0 for pollutants, and (3) using a maximum lag of 14 days for temperature. All statistical analyses were executed in the R platform (version 4.3.0)^45^ using the *mgcv* (version 1.8–42),^34^ *d lnm* (version 2.4.7),^35^ and *Metafor* (version 4.8–0)^43^ packages, with a two-tailed significance level of p-value <0.05.

## Results

Between 2000 and 2019, a total of 10,119,162 NCNE emergency hospitalizations were recorded, with around 14 and 20% due to circulatory and respiratory causes, respectively (Table 1). Daily mean temperatures ranged from 4.9 to 32.4 °C, with a median of 24.8 °C. Over this period, annual average concentrations of PM_2.5_ and NO_2_ declined, while O_3_ levels showed an upward trend. PM_2.5_ and NO_2_ were strongly correlated (Table S2; correlation coefficients 0.69–0.79), both exhibiting low concentrations during the summer and increasing as temperatures dropped (Figure S1). In contrast, the level of O_3_, although also low in summer, peaked between September and October and remained relatively elevated during other months.

**Table 1:** Summary Statistics of Daily Counts of Emergency Hospitalizations and Levels of Environmental Exposures in Hong Kong, 2000–2019^c^

				Percentiles			
Variable	N (%)^a^	Mean (SD)	Minimum	25th	50th	75th	Maximum
Emergency hospitalization							
NCNE	10,119,162 (100.0)	1385 (274)	589	1171	1346	1591	2198
Circulatory	1,371,167 (13.6)	188 (38)	66	159	185	213	330
Respiratory	1,999,813 (19.8)	274 (69)	92	226	262	310	608
Environmental exposure							
Mean temperature, °C	7305 (100.0)	23.6 (5.1)	4.9	19.5	24.8	28.0	32.4
Relative humidity, %	7305 (100.0)	78.3 (10.2)	29.0	74.0	79.0	85.0	99.0
Total rainfall, mm	7305 (100.0)	6.6 (20.7)	0.0	0.0	0.0	1.8	307.1
Wind speed, km/h	7305 (100.0)	22.7 (9.9)	2.5	15.2	22.0	29.0	102.1
PM_2.5_, μg/m^3^	7305 (100.0)	32.0 (20.3)	3.9	16.5	27.0	42.8	179.7
NO_2_, μg/m^3^	7305 (100.0)	53.5 (19.6)	4.1	39.2	50.5	64.4	167.0
O_3_, μg/m^3^ ^b^	7305 (100.0)	56.5 (32.4)	3.8	30.5	51.0	75.5	284.1

a Sum of daily counts (proportion) for hospitalization and number of days (proportion) for environmental exposure.

b Daily maximum 8 h average.

c Abbreviation: SD, standard deviation; NCNE, noncancer nonexternal.

The individual effects of temperature were U-shaped for total NCNE and respiratory hospitalizations, while monotonically inverse for circulatory hospitalizations (Figure 1). With reference to the median temperature of 24.8 °C, RRs (95% CIs) associated with a low temperature of 14.6 °C (5th percentile) were estimated to be 1.000 (0.989, 1.011) for total NCNE, 1.159 (1.142, 1.176) for circulatory, and 1.058 (1.043, 1.072) for respiratory admissions; for a high temperature of 30.0 °C (95th percentile), the corresponding estimates were 1.033 (1.024, 1.042), 0.980 (0.968, 0.993), and 1.025 (1.012, 1.038), respectively. All air pollutants were positively associated with hospitalization risks after adjustment for daily mean temperature and other covariates, with MA03 showing the largest effects and hence being used subsequently to examine the potential modification of air pollutants (Figure 2). The estimated increases in hospitalization risks of different causes per 10-unit increase of MA03 PM_2.5_, NO_2_, and O_3_ were 2.7–7.6%, 3.6–7.4%, and 1.2–6.5%, respectively (Table S3).

**Figure 1:**
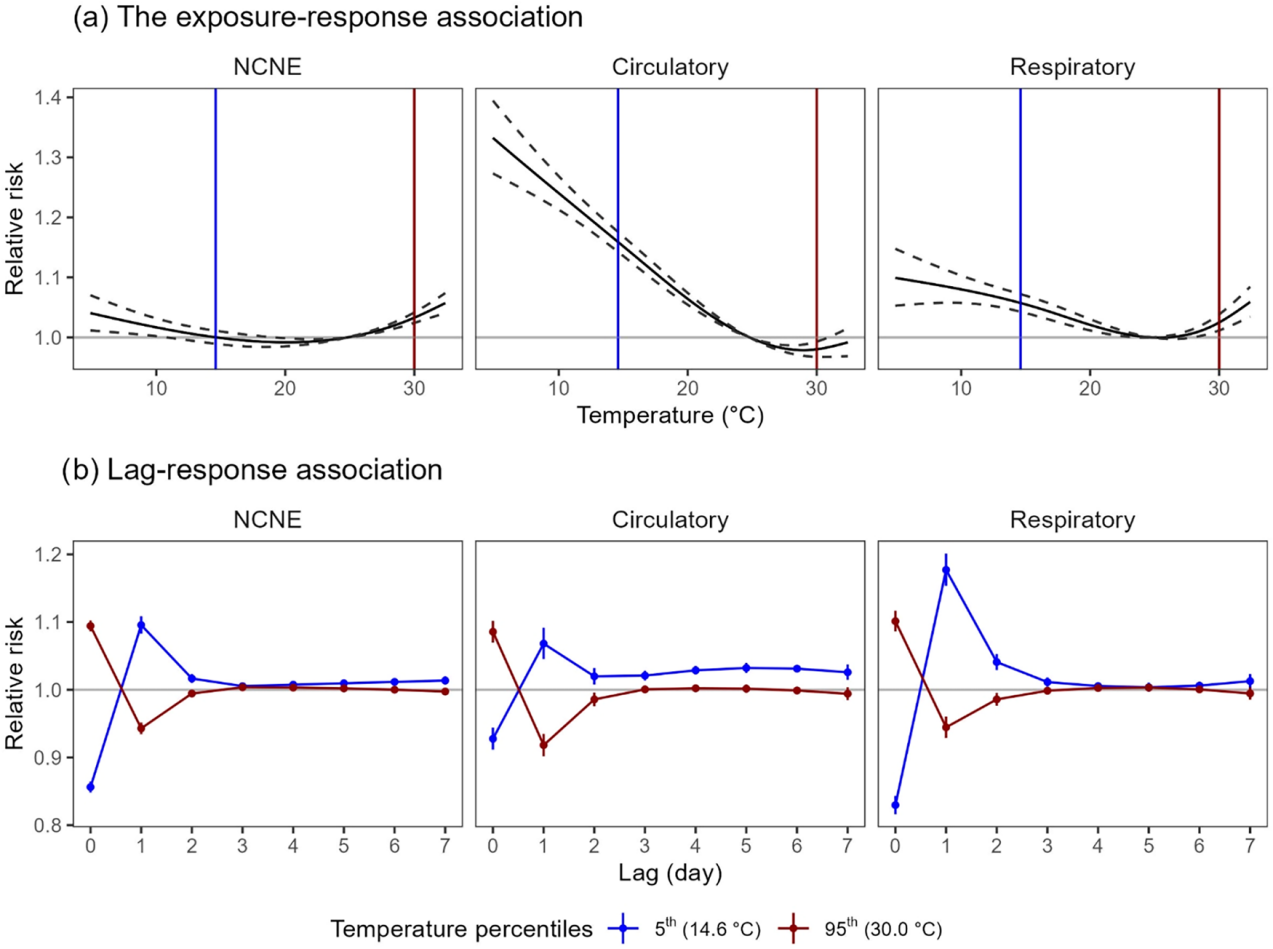
Cumulative exposure–response (a) and lag–response (b) associations between temperature and emergency hospitalizations with a maximum lag of 7 days from the raw models. In panel (a), solid curves represent relative risks, and dashed curves represent 95% confidence intervals; the vertical blue and red lines represent 5th (14.6 °C) and 95th (30 °C) percentiles, respectively. Relative risks are referenced to the median temperature (24.8 °C). Models include the following predictors: temperature crossbasis with a maximum lag of 7 days, MA03 PM2.5, and covariates, i.e., relative humidity, wind speed, rainfall, trend, seasonality, weekdays, and public holidays. Abbreviation: NCNE, noncancer nonexternal; MA03, moving averages of lag 0–3 days.

**Figure 2:**
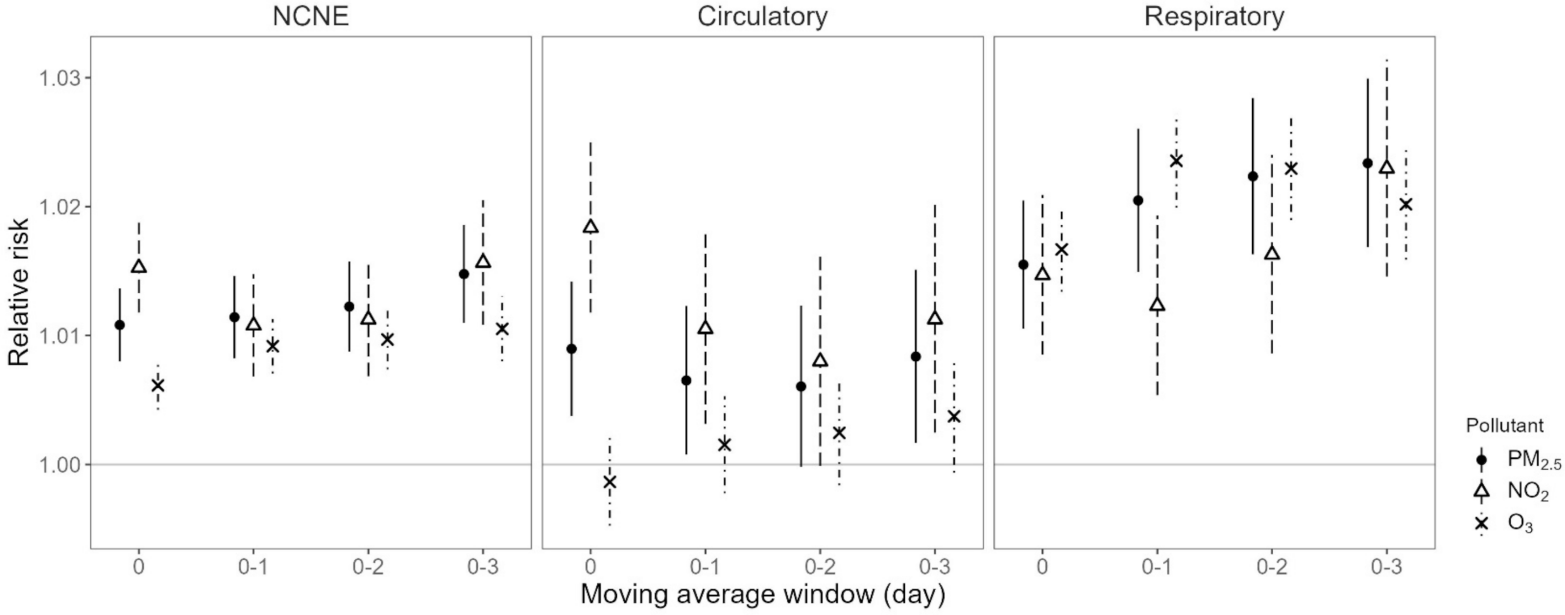
Changes in relative risks of emergency hospitalizations per 10-unit increase in moving average levels of air pollutants from raw models. Points represent relative risks, and line ranges represent 95% confidence intervals. Models include the following predictors: temperature crossbasis with a maximum lag of 7 days, a specific pollutant, and covariates, i.e., relative humidity, wind speed, rainfall, trend, seasonality, weekdays, and public holidays. Abbreviation: NCNE, noncancer nonexternal.

By comparing RRRs for temperature-related emergency hospitalizations across various pollutant thresholds (Tables S4 and S5), we presented models using the 66th percentile cutoff (i.e., PM_2.5_, 36.2 μg/m^3^; NO_2_, 59.1 μg/m^3^; and O_3_, 64.9 μg/m^3^) as the main results, as these consistently yielded significant modification by air pollution for both pollutant-specific and pollutant-load approaches. As shown in Table 2, both low- and high-temperature-related RRs of NCNE admissions significantly increased by 1.6–1.8% with concurrent higher PM_2.5_. Low temperature-related RRs of circulatory admissions have risen by 3.4% (95% CI: 1.0%, 5.8%) with higher PM_2.5_ and 2.7% (0.3%, 5.1%) with higher NO_2_. Conversely, respiratory RRs remained stable between pollutant strata, showing no significant modification.

**Table 2:** Cumulative Temperature-Related Relative Risks (95% Confidence Interval) of Emergency Hospitalizations over a Lag of 0–7 Days by Pollutant Strata^d^

			Low temperature^b^		High temperature^c^	
Hospitalization	Pollutant	strata^a^	RR (95% CI)	RRR (95% CI)	RR (95% CI)	RRR (95% CI)
NCNE	PM_2.5_	Lower	1.000 (0.988, 1.012)	ref	1.016 (1.007, 1.025)	ref
		Higher	1.018 (1.005, 1.031)	1.018 (1.000, 1.036)*	1.033 (1.020, 1.046)	1.016 (1.001, 1.032)*
	NO_2_	Lower	1.003 (0.991, 1.015)	ref	1.019 (1.010, 1.028)	ref
		Higher	1.016 (1.003, 1.029)	1.013 (0.996, 1.031)	1.027 (1.015, 1.040)	1.009 (0.994, 1.024)
	O_3_	Lower	1.006 (0.994, 1.017)	ref	1.018 (1.009, 1.028)	ref
		Higher	1.022 (1.008, 1.035)	1.016 (0.998, 1.034)	1.027 (1.016, 1.037)	1.008 (0.994, 1.022)
Circulatory	PM_2.5_	Lower	1.150 (1.132, 1.168)	ref	0.962 (0.949, 0.976)	ref
		Higher	1.189 (1.170, 1.209)	1.034 (1.011, 1.059)*	0.981 (0.960, 1.001)	1.019 (0.994, 1.044)
	NO_2_	Lower	1.154 (1.136, 1.172)	ref	0.964 (0.950, 0.977)	ref
		Higher	1.186 (1.166, 1.207)	1.028 (1.004, 1.052)*	0.971 (0.952, 0.990)	1.007 (0.983, 1.032)
	O_3_	Lower	1.168 (1.150, 1.187)	ref	0.967 (0.953, 0.981)	ref
		Higher	1.181 (1.160, 1.202)	1.011 (0.987, 1.035)	0.965 (0.950, 0.981)	0.998 (0.977, 1.020)
Respiratory	PM_2.5_	Lower	1.054 (1.037, 1.070)	ref	1.019 (1.004, 1.033)	ref
		Higher	1.063 (1.046, 1.081)	1.009 (0.986, 1.033)	1.019 (0.998, 1.040)	1.000 (0.975, 1.025)
	NO_2_	Lower	1.055 (1.038, 1.071)	ref	1.019 (1.004, 1.034)	ref
		Higher	1.062 (1.044, 1.080)	1.007 (0.984, 1.030)	1.018 (0.998, 1.038)	0.999 (0.975, 1.023)
	O_3_	Lower	1.066 (1.050, 1.083)	ref	1.026 (1.010, 1.041)	ref
		Higher	1.087 (1.068, 1.107)	1.020 (0.996, 1.044)	1.024 (1.007, 1.040)	0.998 (0.976, 1.020)

a MA03 pollutant levels are used, and pollutant strata are defined using their 66th percentile levels (μg/m^3^), i.e., 36.2, 59.1, and 64.9 for PM_2.5_, NO_2_, and O_3_, respectively. Models include the following predictors: temperature crossbasis with a maximum lag of 7 days, a specific pollutant, a product term between the crossbasis and the pollutant strata indicator, and covariates, i.e., relative humidity, wind speed, rainfall, trend, seasonality, weekdays, and public holidays.

b At the 5th percentile temperature (14.6 °C).

c At the 95th percentile temperature (30.0 °C).

d Abbreviation: RR, relative risk; CI, confidence interval; RRR, ratio of relative risk; ref, reference; NCNE, noncancer nonexternal; MA03, moving averages of lag 0–3 days. RR is referenced to the median temperature (24.8 °C). RRR = RR_higher_/RR_lower_. For RRR, * represents p-value <0.05.

In pollutant-load models, more than half of the study days were classified as polluted days, of which two-thirds were in cool seasons (Table S6). Polluted days had 2.2–1.8 times higher mean levels of the three pollutants than unpolluted days. Consistent with findings from pollutant-specific models, both low and high temperature effects on NCNE admissions, as well as low temperature effects on circulatory admissions, were significantly worsened on polluted days, with RRs increased by 1.8, 1.4, and 2.6%, respectively (Table 3). Notably, temperature-related RRs showed significant positive trends as the number of elevated pollutants increased, especially for NCNE (*P*_trend_: 0.003 for low temperature and 0.020 for high temperature) and circulatory (*P*_trend_: 0.002 for low temperature and 0.146 for high temperature) admissions where RRs increased by 3.0–4.0% when all three pollutants were elevated (Figure 3 and Table S7).

**Table 3:** Cumulative Temperature-Related Relative Risks (95% Confidence Interval) of Emergency Hospitalizations over a Lag of 0–7 Days by Pollutant Load^d^

		Low temperature^b^		High temperature^c^	
Hospitalization	Pollutant load^a^	RR (95% CI)	RRR (95% CI)	RR (95% CI)	RRR (95% CI)
NCNE	Unpolluted	0.997 (0.984, 1.009)	ref	1.013 (1.004, 1.023)	ref
	Polluted	1.014 (1.002, 1.026)	1.018 (1.000, 1.036)*	1.027 (1.017, 1.037)	1.014 (1.000, 1.028)*
Circulatory	Unpolluted	1.145 (1.123, 1.167)	ref	0.961 (0.947, 0.976)	ref
	Polluted	1.174 (1.156, 1.192)	1.026 (1.001, 1.051)*	0.967 (0.952, 0.982)	1.006 (0.984, 1.027)
Respiratory	Unpolluted	1.049 (1.030, 1.069)	ref	1.013 (0.998, 1.029)	ref
	Polluted	1.064 (1.048, 1.080)	1.014 (0.990, 1.039)	1.023 (1.008, 1.039)	1.010 (0.988, 1.032)

a MA03 pollutant levels are used, and pollutant strata are defined using their 66th percentile levels (μg/m^3^), i.e., 36.2, 59.1, and 64.9 for PM_2.5_, NO_2_, and O_3_, respectively. Models include the following predictors: temperature crossbasis with a maximum lag of 7 days, a product term between the crossbasis and the pollutant-load indicator, and covariates, i.e., relative humidity, wind speed, rainfall, trend, seasonality, weekdays, and public holidays. Pollutant load is classified as “unpolluted” when 0 pollutant at their higher strata on the same day, and “polluted” when the pollutant count is 1–3.

b At the 5th percentile temperature (14.6 °C).

c At the 95th percentile temperature (30.0 °C).

d Abbreviation: RR, relative risk; CI, confidence interval; RRR, ratio of relative risk; ref, reference; NCNE, noncancer nonexternal; MA03, moving averages of lag 0–3 days. RR is referenced to the median temperature (24.8 °C). RRR = RR_polluted_/RR_unpolluted_. For RRR, * represents p-value <0.05.

**Figure 3:**
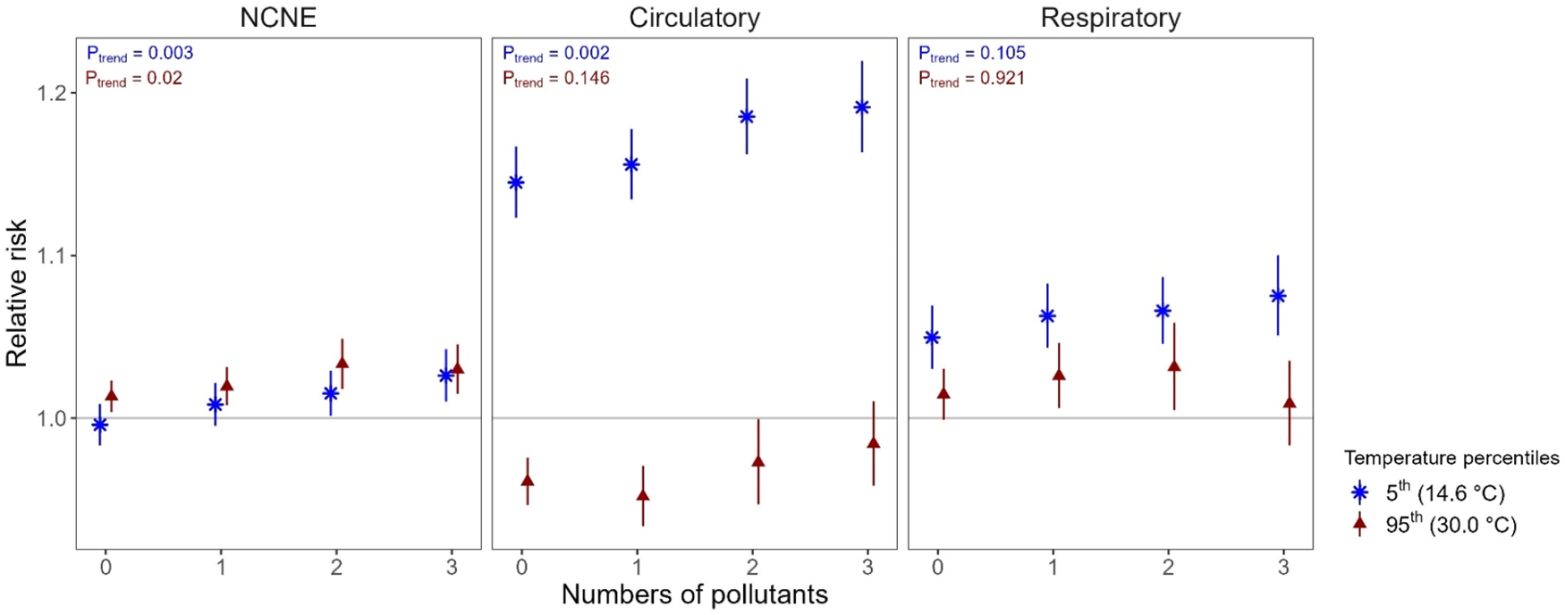
Cumulative temperature-related relative risks of emergency hospitalizations over a lag of 0–7 days by the count of elevated pollutants from pollutant-load models. Points represent relative risks, and line ranges represent 95% confidence intervals. Relative risks are referenced to the median (24.8 °C), respectively. Models include the following predictors: temperature crossbasis with a maximum lag of 7 days, a product term between the crossbasis and the pollutant-load indicator, and covariates, i.e., relative humidity, wind speed, rainfall, trend, seasonality, weekdays, and public holidays. Abbreviation: NCNE, noncancer nonexternal; *P*_trend_, *p*-value for trend.

The observed temperature-ANs (AFs) from the raw models were estimated to be 68,032 (0.7%) NCNE, 44,290 (3.2%) circulatory, and 39,995 (2.0%) respiratory admissions, without considering air pollution modification (Table 4). The real-life scenarios yielded slightly smaller estimates using pollutant-specific and -load models. Under counterfactual low-pollution scenarios, temperature-ANs could be reduced by 54–76% for NCNE, 18–23% for circulatory, and 11–22% for respiratory, compared to the observed estimates, equating to approximate reductions of 37,000–52,000; 8,000–10, 000; and 4000–9000 cases, respectively. Conversely, high-pollution scenarios could increase ANs by 38–67% (≈26,000–46,000 cases) for NCNE, 1–20% for circulatory (≈500–9,000), and 3–10% (≈1,000–4,000) for respiratory admissions.

**Table 4:** Nonoptimal Temperature-Attributable Burdens of Emergency Hospitalizations in Different Models^d^

	Raw model^a^		PM_2.5_-specific model^b^			Pollutant-load model^c^		
Hospitalization	AN (95% eCI)	AF (95% eCI)	Scenario	AN (95% eCI)	AF (95% eCI)	Scenario	AN (95% eCI)	AF (95% eCI)
NCNE	68,032 (35,748, 99,302)	0.7 (0.4, 1.0)	Real-life	60,765 (31,017, 88,261)	0.6 (0.3, 0.9)	Real-life	62,553 (32,004, 93,616)	0.6 (0.3, 0.9)
			Low pollution	31,364 (−3,724, 66,838)	0.3 (−0.0, 0.7)	Low pollution	16,235 (−20,383, 55,998)	0.2 (−0.2, 0.6)
			High pollution	113,753 (65,616, 159,227)	1.1 (0.6, 1.6)	High pollution	93,778 (56,407, 130,509)	0.9 (0.6, 1.3)
Circulatory	44,290 (39,173, 49,205)	3.2 (2.9, 3.6)	Real-life	43,609 (38,436, 48,711)	3.2 (2.8, 3.6)	Real-life	42,118 (36,649, 47,016)	3.1 (2.7, 3.4)
			Low pollution	36,331 (29,882, 41,915)	2.6 (2.2, 3.1)	Low pollution	34,288 (26,559, 42,070)	2.5 (1.9, 3.1)
			High pollution	53,278 (44,456, 61,070)	3.9 (3.2, 4.5)	High pollution	44,774 (38,432, 51,249)	3.3 (2.8, 3.7)
Respiratory	39,995 (32,158, 47,618)	2.0 (1.6, 2.4)	Real-life	38,896 (30,956, 46,599)	1.9 (1.5, 2.3)	Real-life	39,800 (32,279, 47,630)	2.0 (1.6, 2.4)
			Low pollution	35,593 (26,359, 44,645)	1.8 (1.3, 2.2)	Low pollution	31,148 (19,232, 42,511)	1.6 (1.0, 2.1)
			High pollution	41,177 (28,641, 54,269)	2.1 (1.4, 2.7)	High pollution	43,817 (34,679, 52,511)	2.2 (1.7, 2.6)

a Models include the following predictors: temperature crossbasis with a maximum lag of 7 days, PM_2.5_, and covariates, i.e., relative humidity, wind speed, rainfall, trend, seasonality, weekdays, and public holidays.

b Models include the following predictors: temperature crossbasis with a maximum lag of 7 days, PM_2.5_, a product term between the crossbasis and the PM_2.5_ strata indicator, and covariates as in individual models. Attributable burdens are estimated in three scenarios: real-life, using corresponding strata-specific RRs for each day; low pollution, using lower-strata RRs for all days; high pollution, using higher-strata RRs for all days.

c Models include the following predictors: temperature crossbasis with a maximum lag of 7 days, a product term between the crossbasis and the pollutant-load indicator, and covariates as in individual models. Pollutant load is classified as “unpolluted” when 0 pollutant at their higher strata on the same day, and “polluted” when the pollutant count is 1–3. Attributable burdens are estimated in three scenarios: real-life, using corresponding load-specific RRs for each day; low pollution, using unpolluted RRs for all days; high pollution, using polluted RRs for all days.

d Abbreviation: AN, attributable number; AF, attributable fraction; eCI, empirical confidence interval; NCNE, noncancer nonexternal; MA03, moving averages of lag 0–3 days; RR, relative risk. AN and AF are referenced to the median temperature (24.8 °C). MA03 pollutant levels are used, and pollutant strata are defined using their 66th percentile levels (μg/m^3^), i.e., 36.2, 59.1, and 64.9 for PM_2.5_, NO_2_, and O_3_, respectively.

As shown in Table S4, PM_2.5_ consistently amplified the temperature effects on NCNE and circulatory admissions across models using 33rd–85th percentile thresholds for pollutant strata, resulting in up to 2.6% higher temperature-related RRs for NCNE and 3.7% higher for circulatory admissions. NO_2_ was a significant modifier only for low temperature-circulatory associations. Interestingly, evidence for O_3_ modification was limited to models using lower thresholds (25th–50th percentiles), and between-strata RRRs of low temperature decreased as we used higher percentile thresholds. In contrast, the temperature-respiratory associations appeared less affected by concurrent air pollution, given that most of the between-strata RRRs were insignificant. For pollutant-load models, a significant enhancement in temperature effects on polluted days was found for those using 50th–75th percentile thresholds (Table S5). Findings of pollutant-specific models were largely robust across sensitivity analyses, particularly those using 66th and 75th percentiles thresholds, including adjusting for three pollutants simultaneously, using MA0 for pollutants, and extending the temperature lag (Figure S2). For pollutant-load models, pollution’s modification of low temperature effects was also consistently observed (Figure S3).

## Discussion

The study offers novel evidence that concurrent exposure to high levels of major criteria air pollutants (i.e., PM_2.5_, NO_2_, and O_3_) strengthened the short-term associations between nonoptimal temperatures (especially low temperatures) and emergency hospitalizations in Hong Kong. Temperature-related risks of NCNE and circulatory hospitalizations were generally greater in the presence of higher pollutant levels. Notably, PM_2.5_ stood out as an index modifier, consistently increasing temperature-related risks across different thresholds, with generally greater modifying impact than other pollutants and evident modification for both low and high temperatures for NCNE hospitalizations. Additionally, the temperature-related risks are aggravated with increasing numbers of pollutants above predefined exposure thresholds.

### Comparison with Existing Evidence

Both temperature and air pollution have been individually linked to short-term increases in hospitalizations.^3,4,46^ Nevertheless, findings on the heat-related circulatory risks have been mixed across populations.^47^ In our study, heat was linked to fewer circulatory admissions, consistent with findings from certain European,^48^ Chinese,^38,49–51^ and U.S. settings.^52,53^ One explanation is known as harvesting, whereby the most vulnerable individuals may have already been affected or died during earlier hot periods, leaving fewer at risk later.^54^ Liu et al. reported that hypertensive disease admissions often decrease on hot days, likely due to heat-induced vasodilation lowering vascular resistance and cardiac afterload.^50^ Behavioral adaptations, such as staying indoors or using air-conditioning, may further limit heat-related hospitalizations.^55^

Amid the growing research investigating the modifying and interactive effects of temperature and air pollution on health outcomes, most have focused on mortality outcomes with relatively strong evidence for heat and pollutants such as PM and O_3_.^9–13,56^ Studies concerning hospitalizations are limited, many of which treated temperature and season as modifiers for pollution-health associations. PM was the most frequently studied pollutant, and its effects on cardiorespiratory admissions were worsened by low and high temperatures.^14,17–19,21,24,26,57^ Other pollutants, such as O_3_, NO_2_, and SO_2_, were less studied, but their impacts were also found to be heightened at low temperatures.^15,57^ Conversely, only a few studies have examined pollutants’ modification on temperature effects.^24–27^ Our findings show that effect modification by PM_2.5_ and other pollutants was most evident in winter, when low temperatures and elevated pollutant levels frequently co-occur in Hong Kong, particularly affecting NCNE and circulatory admissions. Although we noted a nonsignificant ∼1.9% increase in heat-related circulatory risk with high PM_2.5_, such interactions were less likely because high temperatures in Hong Kong generally coincide with improved dispersion and lower PM_2.5_ levels, limiting the likelihood of combined exposure and thus reducing the ability to detect interaction effects. Consistent with this seasonal pattern, a study in Shenzhen, China, found that low temperatures (10–20 °C) combined with high PM_2.5_ (80–100 μg/m^3^) increased stroke admissions more than low temperatures alone,^26^ and a study in Brisbane, Australia, reported stronger negative associations between temperature and cardiovascular admissions in the presence of high PM_10_.^24^

### Added Values of This Study

The pollutant-specific models allowed the isolation of individual modification effects of PM_2.5_, NO_2_, and O_3_, with PM_2.5_ emerging as a key modifier that warrants targeted environmental and public health interventions. The WHO Air Quality Guidelines recommend a target for 24-h average concentration of PM_2.5_ below 25 μg/m^3^.^29^ However, our analysis indicated that even levels near this guideline could elevate health risks when combined with nonoptimal temperatures, as days with MA03 PM_2.5_ exceeding 21.6 μg/m^3^ (33rd percentile) were associated with 2–3% higher low temperature-related risks of hospitalizations. The pollutant-load models supplemented the pollutant-specific models by accounting for simultaneous exposure to multiple pollutants, capturing the incremental temperature risks as pollutant complexity and quantity increased. For instance, days with lower PM_2.5_ concentrations may still have harmful levels of other pollutants. By comparing days with varying numbers of elevated pollutants to those with none, these models helped reduce misclassification, improve ecological validity, and reinforce the modification effects observed in pollutant-specific models.

While the exact mechanisms behind the observed interaction between air pollution and temperature in relation to the risk of hospitalization remain to be determined, our findings reflect that combined exposure could place greater biological strain than either exposure alone. Temperature and air pollution could act through shared pathways, including systemic inflammation and oxidative stress,^58,59^ endothelial dysfunction,^60,61^ and autonomic imbalance,^62,63^ in increasing disease risk. Cold-induced sympathetic activation and vasoconstriction increase hemoconcentration and clotting tendency, while pollutants exacerbate endothelial injury, platelet activation, and coagulation, creating a strongly pro-thrombotic state that heightens ischemic risk; heat stress triggers vasodilation, dehydration, and hyperventilation, increasing pollutant uptake and intensifying oxidative stress and inflammatory responses.^64,65^ In Hong Kong, air pollution is persistently high in the winter, when PM_2.5_ contains more combustion-related components that could yield greater oxidative injuries per unit mass, allowing biological stress to accumulate under prolonged coexposure.^66^

PM_2.5_ showed a stronger modifying effect than NO_2_, despite their correlation from common sources, such as traffic and industry. PM_2.5_ penetrates deeply into the lungs and enters circulation, carrying redox-active constituents that drive systemic injury, whereas NO_2_ acts predominantly as an airway irritant and asthma sensitizer.^67–69^ Additionally, NO_2_ is short-lived and spatially variable, which increases exposure misclassification and may weaken observed modification.^5^ The modifying effects of O_3_ became less pronounced at higher thresholds, likely because differences in copollutant levels (PM_2.5_ and NO_2_) between low- and high-O_3_ strata were small (strata mean: O_3_, 27.0 vs 62.3 μg/m^3^; PM_2.5_, 32.1 vs 40.1 μg/m^3^; NO_2_, 54.8 vs 61.6 μg/m^3^), especially in winter, making O_3_’s independent modification harder to detect.

Finally, the practical implications are clear. Policymakers can use these findings to inform integrated strategies, such as joint temperature–pollution alerts and targeted interventions during the winter when low temperatures and high pollutants coincide. Patients, particularly older adults and those with pre-existing cardiovascular conditions, can benefit from early warnings of days with extreme temperatures and elevated air pollution and advice to take precautions such as limiting outdoor activities, closely following prescribed medication to ensure stable health condition (e.g., hypertension management), preparation of emergency medications (e.g., blood thinners in case of angina; or corticosteroid inhalers for asthmatic patients). In attributable burden analyses, cleaner air produced much larger reductions in temperature-attributable cases than the increases observed under high-pollution conditions. NCNE admissions were most affected, falling by more than half under low-pollution scenarios but rising by less than two-thirds under high-pollution scenarios, highlighting the disproportionate health benefits of improving air quality.

### Strengths and Limitations

Several strengths of this study could be acknowledged. First, as one of the first studies to examine the effect modification of air pollution on the emergency hospitalization impact of nonoptimal temperature, we utilized an extended time-series data set spanning 20 years with relatively robust statistical power. Second, we estimated temperature-attributable burdens using established methods, comparing observed burdens to counterfactual scenarios—without and with combined air pollution—to understand the potential impact of combined air pollution exposure on these hospitalizations, highlighting the underappreciated indirect health benefits of air quality improvement. However, this study also has limitations. First, as in most studies of this kind, we used citywide averages from fixed monitoring stations, which may not fully reflect individual exposures; such nondifferential misclassification likely adds uncertainty in the estimates rather than bias. Second, Hong Kong’s subtropical climate may limit the generalizability of our findings to regions with different climatic conditions and exposure patterns. Further studies in more diverse settings would be essential to verifying our observations. Third, hospitalizations from external causes (e.g., accidents and suicide) were not included, although they may also be temperature sensitive. Finally, as an ecological study, unmeasured time-varying personal factors (e.g., smoking) could not be controlled, potentially introducing residual confounding.

## Conclusions

Overall, this study provides novel evidence that elevated exposure to criteria air pollutants (PM_2.5_, NO_2_, and O_3_) could aggravate the short-term effects of nonoptimal temperatures on emergency hospitalizations in Hong Kong, especially for those related to low temperatures. Temperature-related risks for NCNE and circulatory hospitalizations increased with higher levels of these pollutants, with PM_2.5_ showing the strongest and most consistent modifying effect across temperature extremes. These findings highlight the need to raise awareness of combined temperature and air pollution risks and strengthen adaptation strategies amid climate change.

## Supplementary Material



## Data Availability

The data of hospital admissions are available from the Hong Kong Hospital Authority, but restrictions apply to the availability of these data, which were used under license for the current study, and so are not publicly available. The data of meteorological variables are available in the Hong Kong Observatory open database https://www.hko.gov.hk/en/abouthko/opendata_intro.htm. The data of air pollutants are available in the Hong Kong Environmental Protection Department open database https://cd.epic.epd.gov.hk/EPICDI/air/station/?lang=en.

## Notes

Tables S1–S8 and Figures S1–S3 with additional details on concentrations of air pollutants at selected percentile thresholds; P correlation coefficients between environmental exposures; RRs (95% CIs) of emergency hospitalizations per 10-unit increase of air pollutants with different moving average windows; RRRs (95% CI) of temperature-related emergency hospitalizations from pollutant-specific models; RRRs (95% CI) of temperature-related emergency hospitalizations from pollutant-burden models; N (%) and mean (SD) of pollutants by numbers of copollutants; temperature-related cumulative RRs (95% CI) over 0–7 days of emergency hospitalizations by numbers of copollutants; difference in mean levels of temperature and air pollutants between pollutant strata; within-year fluctuation of temperature and pollutants in Hong Kong. The horizontal dashed line represents the median level, and results for sensitivity analyses (PDF)
