# Supplementary material for: Aggravated Risks of Emergency Hospitalizations Associated with Temperature amid Elevated Ambient Air Pollution: Evidence from a 20-Year Time-Series Study in Hong Kong
Source: Environ Sci Technol. 2025 Dec 10;59(50):27107–17. doi: 10.1021/acs.est.5c10594 (PMC12750533; doi:10.1021/acs.est.5c10594)

Aggravated risks of emergency hospitalizations associated with temperature amidst elevated ambient air pollution: evidence from a 20-year time-series study in Hong Kong

Yi Tong Guo,[a] Yingxin Li,[a] and Kin Fai Ho[a,b,*], Ka Hung Chan[c,d,*]

[a] JC School of Public Health and Primary Care, The Chinese University of Hong Kong, Hong Kong SAR, China

[b] Institute of Environment, Energy and Sustainability, The Chinese University of Hong Kong, Hong Kong SAR, China

[c] Oxford Global Health, University of Oxford, Oxford, UK

[d] Clinical Trial Service Unit and Epidemiological Studies Unit, Nuffield Department of Population Health, University of Oxford, Oxford, UK

[*] Corresponding author: K.F. Ho: JC School of Public Health and Primary Care, The Chinese University of Hong Kong, Shatin, New Territories, Hong Kong SAR, China. kfho@cuhk.edu.hk;

K.H. Chan: Oxford Global Health, Old Road Campus, University of Oxford, Oxford, UK. peter.chan.oxford@gmail.com

--- Supporting Information ---

Including:

Table S1-S8

Figure S1-S3

Table

Table S1. Concentrations of air pollutants at selected percentile thresholds.

Pollutant	Percentiles					
	25th	33th	50th	66th	75th	85th
$PM_{2.5}$, μg/m^3	18.3	21.6	28.5	36.2	41.9	50.4
NO_2, μg/m^3	41.4	44.9	52.0	59.1	63.6	70.0
O_3, μg/m^3 [a]	34.6	40.3	52.9	64.9	72.4	83.7

[a] Daily maximum 8-hour average.

Table S2. Pearson correlation coefficients between environmental exposures.

		Temperature	$PM_{2.5}$	NO_2	O_3
Whole year	Temperature	1.00	-	-	-
	$PM_{2.5}$	-0.37	1.00	-	-
	NO_2	-0.35	0.77	1.00	-
	O_3	-0.01	0.41	0.29	1.00
Cool season [a]	Temperature	1.00	-	-	-
	$PM_{2.5}$	-0.17	1.00	-	-
	NO_2	-0.06	0.69	1.00	-
	O_3	0.11	0.17	0.01	1.00
Hot season [b]	Temperature	1.00	-	-	-
	$PM_{2.5}$	-0.21	1.00	-	-
	NO_2	-0.21	0.79	1.00	-
	O_3	-0.16	0.63	0.53	1.00

[a] November to December and January to April.
[b] May to October.

Table S3. RRs (95% CIs) of emergency hospitalizations per 10-unit increase of air pollutants with different moving average windows.

Hospitalization	Pollutant	Moving average window (day)			
		0	0-1	0-2	0-3
NCNE	$PM_{2.5}$	1.011 (1.008, 1.014)	1.011 (1.008, 1.015)	1.012 (1.009, 1.016)	1.015 (1.011, 1.019) #
	NO_2	1.015 (1.012, 1.019)	1.011 (1.007, 1.015)	1.011 (1.007, 1.016)	1.016 (1.011, 1.020) #
	O_3	1.006 (1.004, 1.008)	1.009 (1.007, 1.011)	1.010 (1.007, 1.012)	1.011 (1.008, 1.013) #
Circulatory	$PM_{2.5}$	1.009 (1.004, 1.014) #	1.007 (1.001, 1.012)	1.006 (1.000, 1.012)	1.008 (1.002, 1.015)
	NO_2	1.018 (1.012, 1.025) #	1.011 (1.003, 1.018)	1.008 (1.000, 1.016)	1.011 (1.002, 1.020)
	O_3	0.999 (0.995, 1.002)	1.002 (0.998, 1.005)	1.002 (0.998, 1.007)	1.004 (0.999, 1.008) #
Respiratory	$PM_{2.5}$	1.015 (1.011, 1.020)	1.020 (1.015, 1.026)	1.022 (1.016, 1.028)	1.023 (1.017, 1.030) #
	NO_2	1.015 (1.009, 1.021)	1.012 (1.005, 1.019)	1.016 (1.009, 1.024)	1.023 (1.015, 1.031) #
	O_3	1.017 (1.013, 1.020)	1.024 (1.020, 1.027) #	1.023 (1.019, 1.027)	1.020 (1.016, 1.024)

Abbreviation: RR, relative risk; CI, confidence interval; NCNE, non-cancer non-external. #, maximum RR.
Pollutant levels are modelled as linear function separately, adjusted for the crossbasis function of temperature with a maximum lag of 7 days, relative humidity, wind speed, rainfall, trend, seasonality, weekdays, and public holidays.

Table S4. RRRs (95% CI) of temperature-related emergency hospitalizations from pollutant-specific models.

Hospitalization	Pollutant	Pollutant strata threshold (th) [c]					
		25	33	50	66	75	85
Low temperature [a]							
NCNE	$PM_{2.5}$	1.013 (0.992, 1.036)	1.023 (1.004, 1.043) *	1.015 (0.998, 1.033)	1.018 (1.000, 1.036) *	1.025 (1.007, 1.044) *	1.021 (1.002, 1.040) *
	NO_2	1.001 (0.979, 1.023)	1.011 (0.992, 1.031)	1.012 (0.995, 1.030)	1.013 (0.996, 1.031)	1.014 (0.996, 1.032)	1.021 (1.002, 1.041) *
	O_3	1.027 (1.009, 1.046) *	1.027 (1.010, 1.046) *	1.019 (1.002, 1.037) *	1.016 (0.998, 1.034)	1.009 (0.992, 1.028)	0.995 (0.976, 1.015)
Circulatory	$PM_{2.5}$	1.027 (0.992, 1.064)	1.032 (1.003, 1.063) *	1.028 (1.004, 1.053) *	1.034 (1.011, 1.059) *	1.037 (1.013, 1.062) *	1.029 (1.002, 1.056) *
	NO_2	1.030 (0.994, 1.067)	1.030 (1.001, 1.060) *	1.025 (1.000, 1.050) *	1.028 (1.004, 1.052) *	1.025 (1.001, 1.050) *	1.039 (1.012, 1.068) *
	O_3	1.034 (1.006, 1.062) *	1.032 (1.007, 1.059) *	1.015 (0.991, 1.039)	1.011 (0.987, 1.035)	1.012 (0.987, 1.038)	0.994 (0.965, 1.024)
Respiratory	$PM_{2.5}$	1.013 (0.979, 1.048)	1.022 (0.993, 1.051)	1.007 (0.984, 1.031)	1.009 (0.986, 1.033)	1.014 (0.990, 1.038)	1.010 (0.985, 1.037)
	NO_2	0.988 (0.955, 1.022)	0.999 (0.972, 1.028)	1.000 (0.976, 1.024)	1.007 (0.984, 1.030)	1.005 (0.981, 1.030)	1.011 (0.984, 1.038)
	O_3	1.025 (0.998, 1.052)	1.026 (1.002, 1.052) *	1.021 (0.997, 1.045)	1.020 (0.996, 1.044)	1.009 (0.984, 1.035)	0.991 (0.963, 1.021)
High temperature [b]							
NCNE	$PM_{2.5}$	1.011 (0.997, 1.025)	1.013 (0.999, 1.027)	1.015 (1.001, 1.030) *	1.016 (1.001, 1.032) *	1.026 (1.009, 1.044) *	1.022 (1.002, 1.043) *
	NO_2	1.010 (0.996, 1.024)	1.009 (0.995, 1.023)	1.013 (0.999, 1.027)	1.009 (0.994, 1.024)	1.007 (0.991, 1.023)	1.011 (0.992, 1.030)
	O_3	1.008 (0.994, 1.023)	1.012 (0.998, 1.026)	1.009 (0.996, 1.023)	1.008 (0.994, 1.022)	1.002 (0.988, 1.016)	0.999 (0.984, 1.014)
Circulatory	$PM_{2.5}$	1.001 (0.979, 1.024)	1.004 (0.982, 1.027)	1.010 (0.987, 1.033)	1.019 (0.994, 1.044)	1.028 (0.999, 1.057)	1.029 (0.994, 1.065)
	NO_2	1.020 (0.997, 1.043)	1.012 (0.990, 1.035)	1.012 (0.989, 1.035)	1.007 (0.983, 1.032)	1.005 (0.979, 1.032)	1.025 (0.992, 1.059)
	O_3	0.996 (0.973, 1.021)	0.999 (0.977, 1.022)	0.997 (0.976, 1.019)	0.998 (0.977, 1.020)	0.996 (0.973, 1.018)	0.989 (0.965, 1.014)
Respiratory	$PM_{2.5}$	1.008 (0.985, 1.032)	1.015 (0.992, 1.038)	1.004 (0.981, 1.027)	1.000 (0.975, 1.025)	1.010 (0.982, 1.039)	0.985 (0.952, 1.020)
	NO_2	1.001 (0.978, 1.024)	1.007 (0.985, 1.030)	1.003 (0.981, 1.026)	0.999 (0.975, 1.023)	0.990 (0.964, 1.017)	0.999 (0.968, 1.032)
	O_3	0.997 (0.974, 1.021)	0.998 (0.976, 1.021)	0.995 (0.974, 1.017)	0.998 (0.976, 1.020)	0.993 (0.970, 1.016)	0.983 (0.959, 1.008)

Abbreviation: RR, relative risk; CI, confidence interval; RRR, ratio of relative risk; NCNE, non-cancer non-external; FDR, false discovery rate. *, p value < 0.05. RRR between pollutant strata is calculated as RR_{higher}/RR_{lower}.

Models are adjusted for the corresponding pollutant, relative humidity, wind speed, rainfall, trend, seasonality, weekdays, and public holidays.

[a] For low temperature, RR is calculated by comparing temperature at the 5th percentile (14.6°C) vs the median (24.8°C).

[b] For high temperature, RR is calculated by comparing temperature at the 95th percentile (30.0°C) vs the median (24.8°C).

[c] The corresponding concentrations (μg/m^3) of pollutants to the specific thresholds are (in the displayed order): $PM_{2.5}$, 18.3, 21.6, 28.5, 36.2, 41.9, and 50.4; NO_2, 41.4, 44.9, 52.0, 59.1, 63.6, and 70.0; O_3, 34.6, 40.3, 52.9, 64.9, 72.4, and 83.7.

Table S5. RRRs (95% CI) of temperature-related emergency hospitalizations from pollutant-burden models.

**Hospitalization**	**Pollutant strata threshold (th)** [c]					
	25	33	50	66	75	85
Low temperature [a]						
NCNE	0.985 (0.924, 1.051)	1.021 (0.987, 1.056)	1.016 (0.996, 1.036)	1.018 (1.000, 1.036) *	1.016 (0.998, 1.033)	1.012 (0.994, 1.029)
Circulatory	1.044 (0.924, 1.181)	1.022 (0.962, 1.087)	1.017 (0.987, 1.049)	1.026 (1.001, 1.051) *	1.027 (1.004, 1.051) *	1.020 (0.996, 1.044)
Respiratory	0.895 (0.798, 1.004)	1.025 (0.966, 1.086)	1.003 (0.974, 1.033)	1.014 (0.990, 1.039)	1.006 (0.984, 1.030)	1.003 (0.980, 1.026)
High temperature [b]						
NCNE	1.017 (0.998, 1.036)	1.013 (0.997, 1.028)	1.014 (1.000, 1.028) *	1.014 (1.000, 1.028) *	1.011 (0.997, 1.025)	1.012 (0.998, 1.027)
Circulatory	1.010 (0.977, 1.044)	1.000 (0.974, 1.026)	1.003 (0.980, 1.026)	1.006 (0.984, 1.027)	1.003 (0.982, 1.025)	1.004 (0.981, 1.027)
Respiratory	1.017 (0.985, 1.050)	1.002 (0.977, 1.028)	1.002 (0.979, 1.025)	1.010 (0.988, 1.032)	1.003 (0.981, 1.025)	0.996 (0.973, 1.019)

Abbreviation: RR, relative risk; CI, confidence interval; RRR, ratio of relative risk; NCNE, non-cancer non-external; FDR, false discovery rate. *, FDR p value < 0.05. RRR is calculated as $RR_{1\text{-}3}/RR_0$.

Models are adjusted for relative humidity, wind speed, rainfall, trend, seasonality, weekdays, and public holidays.

[a] For low temperature, RR is calculated by comparing temperature at the 5th percentile (14.6°C) vs the median (24.8°C).

[b] For high temperature, RR is calculated by comparing temperature at the 95th percentile (30.0°C) vs the median (24.8°C).

[c] The corresponding concentrations (μg/m^3) of pollutants to the specific thresholds are (in the displayed order): $PM_{2.5}$, 18.3, 21.6, 28.5, 36.2, 41.9, and 50.4; NO_2, 41.4, 44.9, 52.0, 59.1, 63.6, and 70.0; O_3, 34.6, 40.3, 52.9, 64.9, 72.4, and 83.7.

Table S6. N (%) and mean (SD) of pollutants by numbers of co-pollutants (threshold percentile: 66th).

# of elevated pollutants [a]	N (%)	Mean (SD), μg/m^3		
		$PM_{2.5}$	NO_2	O_3
2-strata				
0 (unpolluted)	3223 (44.1)	19.1 (7.5)	42.0 (9.1)	37.7 (13.0)
1-3 (polluted)	4079 (55.8)	42.1 (16.7)	62.6 (14.9)	71.3 (25.8)
4-strata				
0	3223 (44.1)	19.1 (7.5)	42.0 (9.1)	37.7 (13.0)
1	1675 (22.9)	28.9 (8.8)	51.4 (10.2)	70.3 (27.4)
2	1441 (19.7)	47.8 (13.0)	67.8 (12.5)	62.0 (22.3)
3	963 (13.2)	56.7 (15.1)	74.2 (11.4)	87.0 (20.1)

Abbreviation: SD, standard deviation.

[a] The 0-3 day moving averages of pollutants are classified into lower and higher levels using the 66th percentile values (μg/m^3) as the threshold, i.e., 36.2, 59.1 and 64.9 for $PM_{2.5}$, NO_2, and O_3, respectively. Pollutants at their higher levels are counted.

Table S7. Temperature-related cumulative RRs (95% CI) over 0-7 days of emergency hospitalizations by numbers of co-pollutants (threshold percentile: 66th).

Hospitalization	# of pollutants [a]	Low temperature		High temperature	
		RR (95% CI) [b]	p-value for trend	RR (95% CI) [c]	p-value for trend
NCNE	0	0.996 (0.983, 1.009)	0.003	1.013 (1.004, 1.023)	0.020
	1	1.008 (0.995, 1.022)		1.019 (1.008, 1.031)	
	2	1.015 (1.001, 1.029)		1.033 (1.018, 1.049)	
	3	1.026 (1.010, 1.042)		1.030 (1.015, 1.045)	
Circulatory	0	1.145 (1.123, 1.167)	0.002	0.961 (0.947, 0.976)	0.146
	1	1.156 (1.134, 1.178)		0.952 (0.933, 0.971)	
	2	1.185 (1.162, 1.209)		0.973 (0.947, 0.999)	
	3	1.191 (1.163, 1.220)		0.984 (0.959, 1.010)	
Respiratory	0	1.050 (1.030, 1.069)	0.105	1.015 (0.999, 1.030)	0.921
	1	1.063 (1.043, 1.083)		1.026 (1.006, 1.046)	
	2	1.066 (1.046, 1.087)		1.031 (1.005, 1.059)	
	3	1.075 (1.051, 1.100)		1.009 (0.983, 1.035)	

Abbreviation: RR, relative risk; CI, confidence interval; NCNE, non-cancer non-external; FDR, false discovery rate. P-value for trend is computed using meta regression with inverse-variance weighting method.
Models are adjusted for relative humidity, wind speed, rainfall, trend, seasonality, weekdays, and public holidays.
[a] The 0-3 day moving averages of pollutants are classified into lower and higher levels using the 66th percentile values (μg/m^3) as the threshold, i.e., 36.2, 59.1 and 64.9 for $PM_{2.5}$, NO_2, and O_3, respectively. Pollutants at their higher levels are counted.
[b] RR is calculated by comparing temperature at the 5th percentile (14.6°C) vs the median (24.8°C).
[c] RR is calculated by comparing temperature at the 95th percentile (30°C) vs the median (24.8°C).

Table S8. Difference in mean levels of temperature and air pollutants between pollutant strata.

Pollutant	Exposure	Cool season						Hot season					
		25th	33th	50th	66th	75th	85th	25th	33th	50th	66th	75th	85th
$PM_{2.5}$	Temperature, °C	-1.7	-1.7	-1.7	-1.6	-1.5	-1.4	-1.2	-1.2	-1.1	-1.0	-1.0	-0.9
	$PM_{2.5}$, μg/m^3	24.5	23.6	23.7	25.9	28.0	32.1	22.1	24.1	28.0	32.8	36.4	42.7
	NO_2, μg/m^3	20.7	19.3	18.5	19.0	19.9	21.5	19.0	20.1	22.3	24.4	25.8	28.7
	O_3, μg/m^3	11.2	9.0	5.0	4.4	5.2	6.6	36.2	38.7	39.2	41.2	43.1	46.7
NO_2	Temperature, °C	-1.7	-1.6	-1.2	-1.1	-0.9	-1.0	-1.3	-1.2	-1.1	-0.7	-0.6	-0.5
	$PM_{2.5}$, μg/m^3	20.5	19.8	19.9	21.0	22.2	24.6	19.0	20.5	24.5	27.2	30.8	35.4
	NO_2, μg/m^3	24.8	23.6	22.6	23.1	24.3	27.2	22.2	23.0	25.5	28.6	31.0	35.3
	O_3, μg/m^3	-1.3	-2.1	0.4	3.2	4.6	5.9	28.7	30.0	33.4	36.4	39.0	44.6
O_3	Temperature, °C	1.1	1.0	0.8	1.3	1.6	1.8	-1.0	-1.1	-1.1	-1.1	-1.0	-0.9
	$PM_{2.5}$, μg/m^3	7.9	7.4	6.7	4.4	1.9	0.5	15.9	17.6	19.4	20.2	20.9	21.2
	NO_2, μg/m^3	6.8	5.9	4.7	1.7	-0.7	-3.0	13.8	14.8	15.4	15.5	15.7	15.8
	O_3, μg/m^3	35.3	34.3	33.3	34.8	37.5	44.1	45.5	48.2	52.8	56.5	59.0	63.6

Difference in mean = $Mean_{higher}$ - $Mean_{lower}$.
The concentrations of pollutants (in μg/m^3) corresponding to the 25th~85th percentiles are: $PM_{2.5}$, 18.3, 21.6, 28.5, 36.2, 41.9, and 50.4; NO_2, 41.4, 44.9, 52.0, 59.1, 63.6, and 70.0; O_3, 34.6, 40.3, 52.9, 64.9, 72.4, and 83.7.
Cool season, Nov - Apr; hot season, May to Oct.

Figure

Figure S1. Within-year fluctuation of temperature and pollutants in Hong Kong. The horizontal dashed line represents the median level.

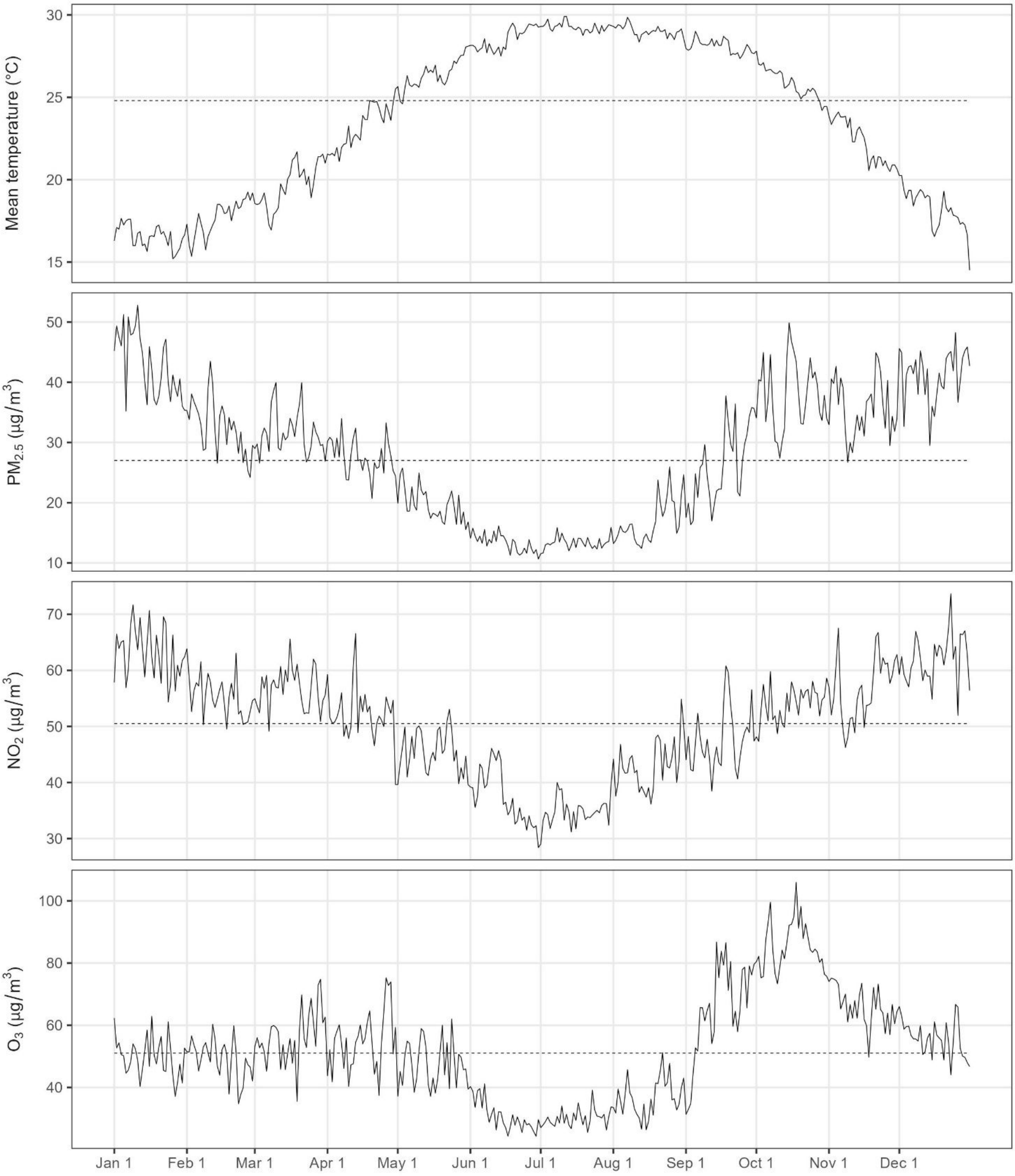

Figure S2. RRRs of temperature-related emergency hospitalizations from pollutant-specific models and sensitivity analysis. RRRs are calculated by comparing higher to lower pollutant strata. Abbreviation: NCNE, non-cancer non-external; MA, moving average.

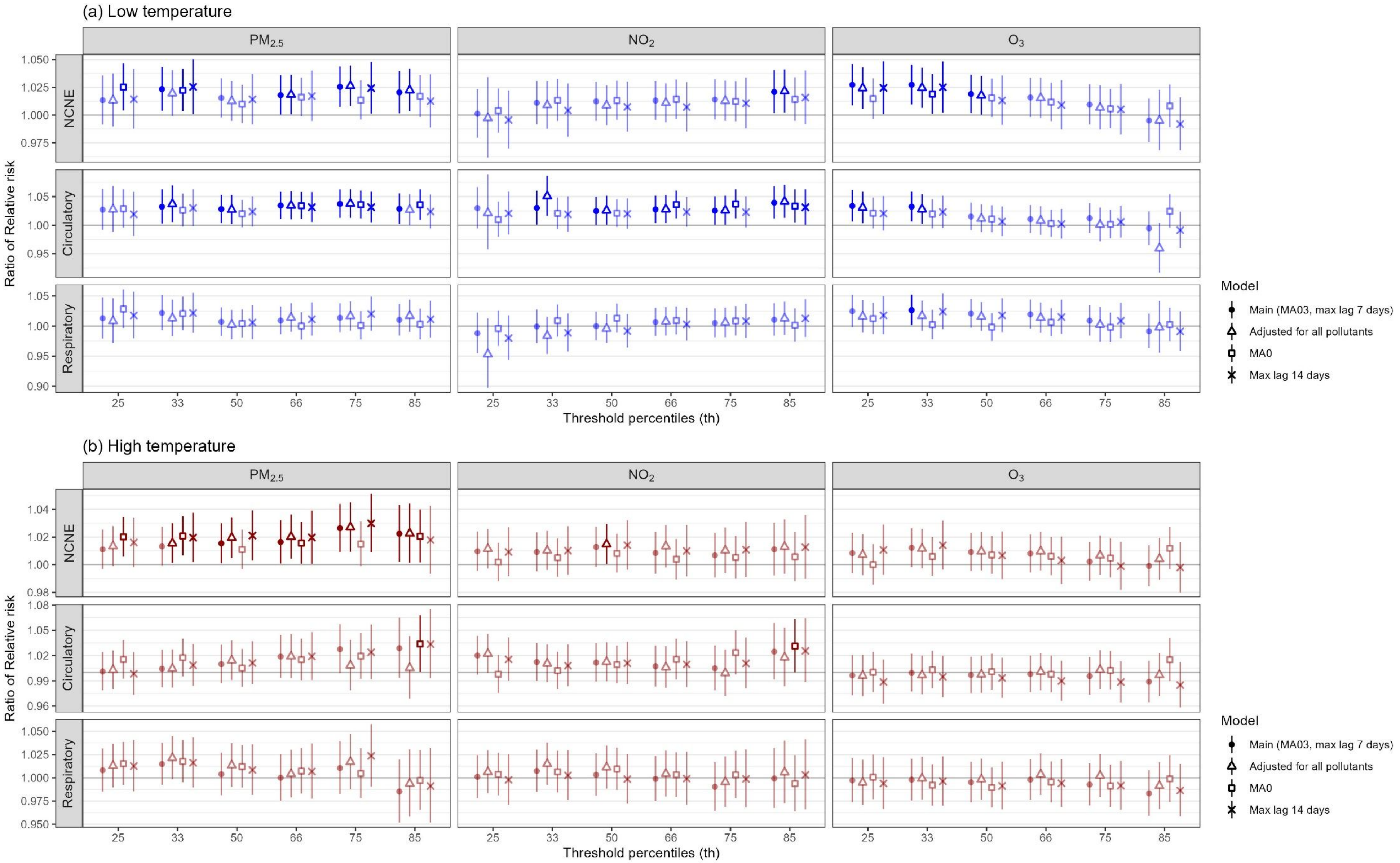

Figure S3. RRRs of temperature-related emergency hospitalizations from pollutant-load models and sensitivity analysis. RRRs are calculated by comparing polluted days to unpolluted days. Abbreviation: NCNE, non-cancer non-external; MA, moving average.

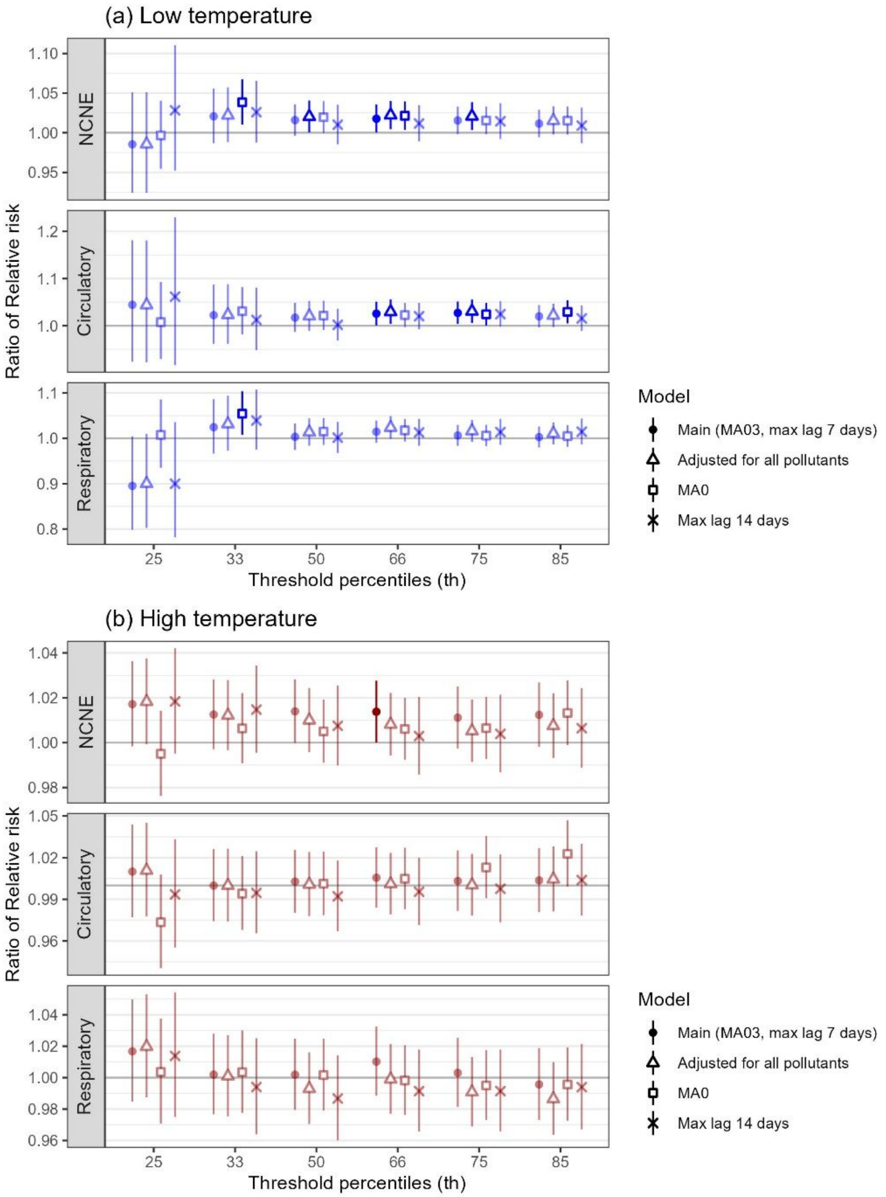

Supplement: Supplementary file 1 [file es5c10594_si_001.pdf]
